# Inhibition of γ-Secretase Leads to an Increase in Presenilin-1

**DOI:** 10.1007/s12035-017-0705-1

**Published:** 2017-08-16

**Authors:** Aitana Sogorb-Esteve, María-Salud García-Ayllón, Marta Llansola, Vicente Felipo, Kaj Blennow, Javier Sáez-Valero

**Affiliations:** 1 Instituto de Neurociencias de Alicante, Universidad Miguel Hernández-CSIC, Av. Ramón y Cajal s/n, 03550 Sant Joan d’Alacant, Spain; 2 Centro de Investigación Biomédica en Red sobre Enfermedades Neurodegenerativas (CIBERNED), Sant Joan d’Alacant, Spain; 3 Unidad de Investigación, Hospital General Universitario de Elche, FISABIO, 03203 Elche, Spain; 4 Laboratory of Neurobiology, Fundación Centro de Investigación Príncipe Felipe, Valencia, Spain; 5 Clinical Neurochemistry Laboratory, Sahlgrenska University Hospital, Mölndal, Sweden; 6 Institute of Neuroscience and Physiology, University of Gothenburg, Mölndal Campus, Sweden

**Keywords:** Alzheimer’s disease, Presenilin-1, γ-Secretase inhibitor, Therapy

## Abstract

γ-Secretase inhibitors (GSIs) are potential therapeutic agents for Alzheimer’s disease (AD); however, trials have proven disappointing. We addressed the possibility that γ-secretase inhibition can provoke a rebound effect, elevating the levels of the catalytic γ-secretase subunit, presenilin-1 (PS1). Acute treatment of SH-SY5Y cells with the GSI LY-374973 (*N*-[*N*-(3,5-difluorophenacetyl)-l-alanyl]-*S*-phenylglycine *t*-butyl ester, DAPT) augments PS1, in parallel with increases in other γ-secretase subunits nicastrin, presenilin enhancer 2, and anterior pharynx-defective 1, yet with no increase in messenger RNA expression. Over-expression of the C-terminal fragment (CTF) of APP, C99, also triggered an increase in PS1. Similar increases in PS1 were evident in primary neurons treated repeatedly (4 days) with DAPT or with the GSI BMS-708163 (avagacestat). Likewise, rats examined after 21 days administered with avagacestat (40 mg/kg/day) had more brain PS1. Sustained γ-secretase inhibition did not exert a long-term effect on PS1 activity, evident through the decrease in CTFs of APP and ApoER2. Prolonged avagacestat treatment of rats produced a subtle impairment in anxiety-like behavior. The rebound increase in PS1 in response to GSIs must be taken into consideration for future drug development.

**Electronic supplementary material:**

The online version of this article (doi:10.1007/s12035-017-0705-1) contains supplementary material, which is available to authorized users.

## Introduction

Alzheimer’s disease (AD) is the most common dementia in the elderly, and it is characterized by extracellular deposits of aggregated β-amyloid (Aβ) peptides and accumulation of intracellular tangles of the abnormally hyperphosphorylated microtubule-associated protein tau (P-tau) [1]. According to the amyloid cascade hypothesis, which is the most prevalent view on AD pathogenesis, the disease pathophysiology is triggered by an excess of neurotoxic Aβ peptides, potentially in combination with other genetics and risk factors [2]. Drug candidates targeting Aβ have dominated AD drug development programs for the past three decades [3], and accordingly, targets for each individual step in this cascade have been developed, with β/γ-secretase inhibitors representing one particular opportunity for front-line therapy.

The Aβ peptide is generated by successive proteolytic processing of the amyloid precursor protein (APP) by secretases. APP is a type I transmembrane spanning glycoprotein that is first processed by either α- or β-secretase, followed by γ-secretase cleavage. β- and γ-secretase cleavage generate Aβ peptides of variable amino acid length, being the most abundant the Aβ40 peptide [4] while Aβ42 appears to be the most amyloidogenic [5]. The major neuronal β-secretase is the beta-site APP-cleaving enzyme 1 (BACE1) [6], while the γ-secretase enzyme complex contains four essential subunits: presenilin-1 (or presenilin-2), nicastrin, anterior pharynx-defective 1 (APH1), and presenilin enhancer 2 (PEN2) [7]. γ-Secretase acts an aspartyl protease, which catalytic core is presenilin-1 (PS1), being its dysfunction associated with the pathological development of AD [8]. Thus, compounds that inhibit γ-secretase, targeting PS1, are potential therapeutic agents for AD.

Preclinical studies clearly established that γ-secretase inhibitors (GSIs) reduce brain Aβ in rodent models and also reverse Aβ-induced cognitive deficits in the AD Tg2576 mice [9]. However, the therapeutic effect of such drugs in humans has fallen below expectation, with no demonstrated efficacy in clinical trials and even impaired cognitive function in long-term treated subjects [10]. Problems of tolerability and dose-limiting effects during clinical trials with GSIs may have compromised target engagement for arriving to the minimum extent of Aβ lowering for significant cognitive benefit in AD patients (discussed in Toyn and Ahlijanian [11]). On the other hand, a paradoxical increase of plasma Aβ levels has been observed upon chronic treatment with a classical GSI in transgenic animal [12]. Treatment of transgenic mice and humans with other GSIs, including compounds involved in clinical trials, may cause late rebound effects on plasma Aβ levels [13–15]. These changes may be illustrative of a rebound effect in reaction to inhibition by a GSI-based therapy. To decipher why current GSIs fail to improve the disease state may help to optimize future drug development.

Upregulation of enzyme isoforms [16, 17], and also of the specific enzyme targeted by the drug [18–20], is not an uncommon phenomenon in reaction to inhibition, although to our knowledge, this possible effect remains unexplored in terms of GSI treatment. Interestingly, we recently reported that an increase in acetylcholinesterase could block γ-secretase activity and that this inhibition initiates a feedback process that leads to a rebound effect, elevating PS1 levels [21]. Here, we tested how GSIs affect PS1 levels in cellular and animal models. As such, we provide evidence that γ-secretase inhibition could provoke a rebound increase in PS1, which may be of particular importance for the design of specific AD therapies based on GSIs and related drugs.

## Materials and Methods

### Cell Cultures and Pharmacological Treatment with GSIs

SH-SY5Y cells (700,000 cells/well) were grown in six-well plates for 24 h in Dulbecco’s modified Eagle’s medium (DMEM) + GlutaMAX™ (Gibco^®^ Life Technologies, Paisley, UK) supplemented with 10% fetal bovine serum (FBS; Gibco) and 100 μg/mL penicillin/streptomycin (Gibco). The cells were treated with 5 μM of γ-secretase inhibitor LY-374973: *N*-[*N*-(3,5-difluorophenacetyl)-l-alanyl]-*S*-phenylglycine *t*-butyl ester (DAPT; Calbiochem^®^, Merck KGaA) or the dimethyl sulfoxide (DMSO) vehicle alone. Following an 18-h treatment, the cells were washed twice with cold phosphate-buffered saline (PBS) and resuspended in 100 μL of ice-cold extraction buffer supplemented with a cocktail of protease inhibitors: 50 mM Tris-HCl (pH 7.4), 150 mM NaCl, 5 mM EDTA, 1% (*w*/*v*) Nonidet P-40, and 0.5% (*w*/*v*) Triton X-100. Cell lysates were sonicated and centrifuged for 1 h at 70,000×*g* and 4 °C, and the extracts were frozen at −80 °C for future analysis.

For some experiments, SH-SY5Y cells were transfected with 4 μg of a construct that encodes the C-terminal 99 amino acids of APP (amino acids 597–695), extending from the β-secretase cleavage site to the C-terminus (a generous gift from David H. Small). A pCI *empty* vector (Promega) served as the negative control. These cells (7 × 10^5^ cells/well) were then seeded on 35-mm tissue culture dishes and transfected using Lipofectamine^®^ 2000 (Thermo Scientific™) according to the manufacturer’s instructions. After 2 days in culture, the cells and culture supernatants were harvested separately, and the cell culture supernatants were cleared by centrifugation at 1000×*g* for 10 min at 4 °C. The cells were then washed with PBS and solubilized as described above. C-terminal fragment of APP (APP-CTF) levels were assayed in Western blots to determine transfection efficiency.

To culture primary cortical neurons, cortical lobes from E16.5 mice embryos were trypsinized and dissociated in Hank’s balanced salt solution (Life Technologies). Neurons were plated onto 35-mm dishes (1.3 × 10^6^ cells/dish) and maintained in Neurobasal medium (Invitrogen) containing B27 supplement (Gibco BRL), 100 IU/mL penicillin, 100 μg/mL streptomycin, and 2 mM glutamine. After 7 days in culture, the cortical neurons were treated with 2 μM of DAPT or the GSI avagacestat (BMS-708163; from Bristol-Myers Squibb) for four consecutive days and analyzed on day 5, 18 h after the last dose. The cells were washed with PBS and solubilized as described above.

Cell viability was measured using the tetrazolium assay (MTS; CellTiter 96^®^ AQueous Assay, Promega) according to the manufacturer’s instructions. Cells were cultured in 96-well plates and treated with GSIs as previously stated. MTS was added after GSI treatment, cells were incubated for 4 h, and then viability was determined by measuring the absorbance at 490 nm in a microplate reader (Infinite M200, Tecan).

### Animals and Tissue Preparation

All animal procedures were approved by the Animal Care and Use Committees at the Universidad Miguel Hernández and by Centro Principe Felipe (2016A/SC/PEA/00127). Wistar male rats that weighed 250–300 g at the beginning of GSI administration were used. The rats were orally administered the avagacestat (40 mg/kg) or vehicle alone (polyethylene glycol) using a single or once-a-day dose for 4 or 21 days (*n* = 10 for each group), and they were sacrificed ~ 4 h after the final administration of avagacestat. Cerebrospinal fluid (CSF) samples (50–60 μL) were collected by cisternal puncture with a needle inserted in the suboccipital region through the atlanto-occipital membrane, with a single incision into the subarachnoid space [22]. CSF samples were centrifuged at 1000×*g* for 10 min at 4 °C, and the supernatants were stored at −80 °C. In addition, the rat’s brain was removed and their cerebral cortices were dissected out and stored at −80 °C. Hemi-cortices were thawed slowly at 4 °C and homogenized (10% *w*/*v*) in extraction buffer: 50 mM Tris-HCl (pH 7.4)/500 mM NaCl/5 mM EDTA/1% (*w*/*v*) Nonidet P-40/0.5% (*w*/*v*) Triton X-100, supplemented with a cocktail of protease inhibitors [23]. The homogenates were sonicated and centrifuged, as indicated above, and the supernatants were collected and frozen at −80 °C. Protein concentrations were determined using the bicinchoninic acid method (Pierce, Rockford, IL, USA). The other hemi-cortices were reserved for messenger RNA (mRNA) analysis (see below).

### Western Blotting

Cell (20 μg) and brain extracts (40 μg) and CSF samples (30 μL) were resolved by sodium dodecyl sulfate-polyacrylamide gel electrophoresis (SDS-PAGE) under fully reducing conditions. Samples were denatured at 50 °C for 15 min to analyze PS1 or, alternatively, at 98 °C for 5 min for other proteins. The proteins separated were transferred to nitrocellulose membranes (Schleicher and Schuell Bioscience GmbH) and probed with a PS1 antibody raised against amino acids 1–20 (antibody 98/1; see Evin et al. [24]). Protein extracts from cell cultures were also probed for other γ-secretase subunits using the following antibodies: mouse anti-nicastrin (Millipore), rabbit anti-PEN2 (Sigma), and rabbit anti-APH1 (which recognizes both the APH1A and APH1B homologs; Sigma).

Brain extracts were also assayed for the CTF of APP or ApoER2 using the monoclonal anti-APP C-terminal antibody C1-6.1 (Covance) or a polyclonal antiserum against the C-terminal of ApoER2 (Abcam). Alternatively, the anti-APP monoclonal antibody 6E10 (Covance) was used. A rabbit anti-glyceraldehyde 3-phosphate dehydrogenase (GAPDH) antibody (Abcam) was used as a loading control. Western blots for different antibodies were performed separately to avoid re-probing the membranes. Antibody binding was detected with the corresponding conjugated secondary antibody (IRDye 680CW goat anti-mouse and IRDye 800RD goat anti-rabbit; LI-COR Biosciences) and visualized on an Odyssey CLx Infrared Imaging System (LI-COR Biosciences). Densitometric quantification of the signal from immunoreactive bands was obtained using LI-COR software (Image Studio Lite).

### RNA Isolation and the Analysis of γ-Secretase Subunit Transcripts by qRT-PCR

The transcripts encoding PS1, nicastrin, PEN2, and two forms of APH1 (APH1A and APH1B) were assayed. The total RNA from rat brain hemi-cortices, SH-SY5Y cells, and mouse cortical neurons was isolated with the TRIzol^®^ Reagent using the PureLink™ Micro-to-Midi Total RNA Purification System (Invitrogen™ Life Technologies), following the manufacturer’s instructions. First-strand complementary DNAs (cDNAs) were synthesized by reverse transcription of 1.5 of total RNA using the High Capacity cDNA Reverse Transcription Kit (Applied Biosystems; Life Technologies) according to the manufacturer’s instructions. Quantitative PCR amplification was performed using a StepOne™ Real-Time PCR System (Applied Biosystems) and TaqMan PCR Master Mix with specific TaqMan Gene Expression Assays: Hs00997789 for PS1, Hs00950933_m1 for nicastrin, Hs00708570_s1 for PEN2, HS00211268_m1 for APH1A, and Hs0029911_m1 for APH1B on SH-SY5Y cell RNA; Mm00501184_m1 for PS1 on mouse cortical neuron RNA; and Rn00569763_m1 for PS1 on rat brain hemi-cortex RNA. GAPDH was amplified as a housekeeping marker (Hs03929097 for SH-SY5Y cells, Mm99999915_g1 for mouse cortical neurons, and Rn014626662_g1 for rat brain hemi-cortices), and the transcript levels were calculated relative to GAPDH using the comparative 2^−ΔCt^ method.

### Behavioral Studies

The Y-maze alternation, active avoidance, and beam walking tests were performed to analyze memory and learning functions, as well as motor coordination. The tests were performed 2–4 h after the final administration of avagacestat.

#### Y-Maze Novel Spatial Recognition Memory

This test is based on the rodents’ natural curiosity to explore novel areas, and the rats were tested as described elsewhere [25]. Briefly, rats were placed into one of the arms of the Y-maze (start arm) and allowed to explore the maze with one of the arms closed for 3 min (training trial). After a 30-min inter-trial interval, the rats were returned to the Y-maze, placing them in the start arm, and then the rats were allowed to freely explore all three arms of the maze for 3 min (test trial). The number of entries into and the time spent in each arm were registered manually by an observer blinded to the rat’s treatment. The discrimination ratio is a measure of the preference for the novel arm over the familiar (old) arm, calculated as the Time spent in Novel / Time spent in the (Novel + Old).

#### Active Avoidance

The active avoidance task is designed to test the ability of the rats to avoid an aversive event by first learning to perform a specific behavior in response to a stimulus. The test was performed on a single day and involved 50 trials per animal, as described previously [26].

#### Beam Walking Test

The beam walking test assesses deficits in fine motor coordination [27], although it is also a useful assay to test for anxiety-like behavior [28] as it also causes some anxiety in the animal. Motor coordination was tested on a 1-m-long wooden stick (20 mm in diameter) situated approximately 1 m above the ground as described elsewhere [29]. The number of slips (foot faults) and the latency to cross (the time spent on the apparatus as an estimate of anxiety) are scored.

### Statistical Analysis

All data were analyzed using SigmaStat (version 3.5; Systat Software, Inc.), determining exact *p* values by applying a Student’s *t* test (two-tailed) or the Mann-Whitney rank-sum test, when normality was rejected. The results are presented as the means ± SEM.

## Results

### Inhibition of γ-Secretase by the GSI DAPT Increases the PS1 in SH-SY5Y and Primary Neuronal Cultures

**Fig. 1:**
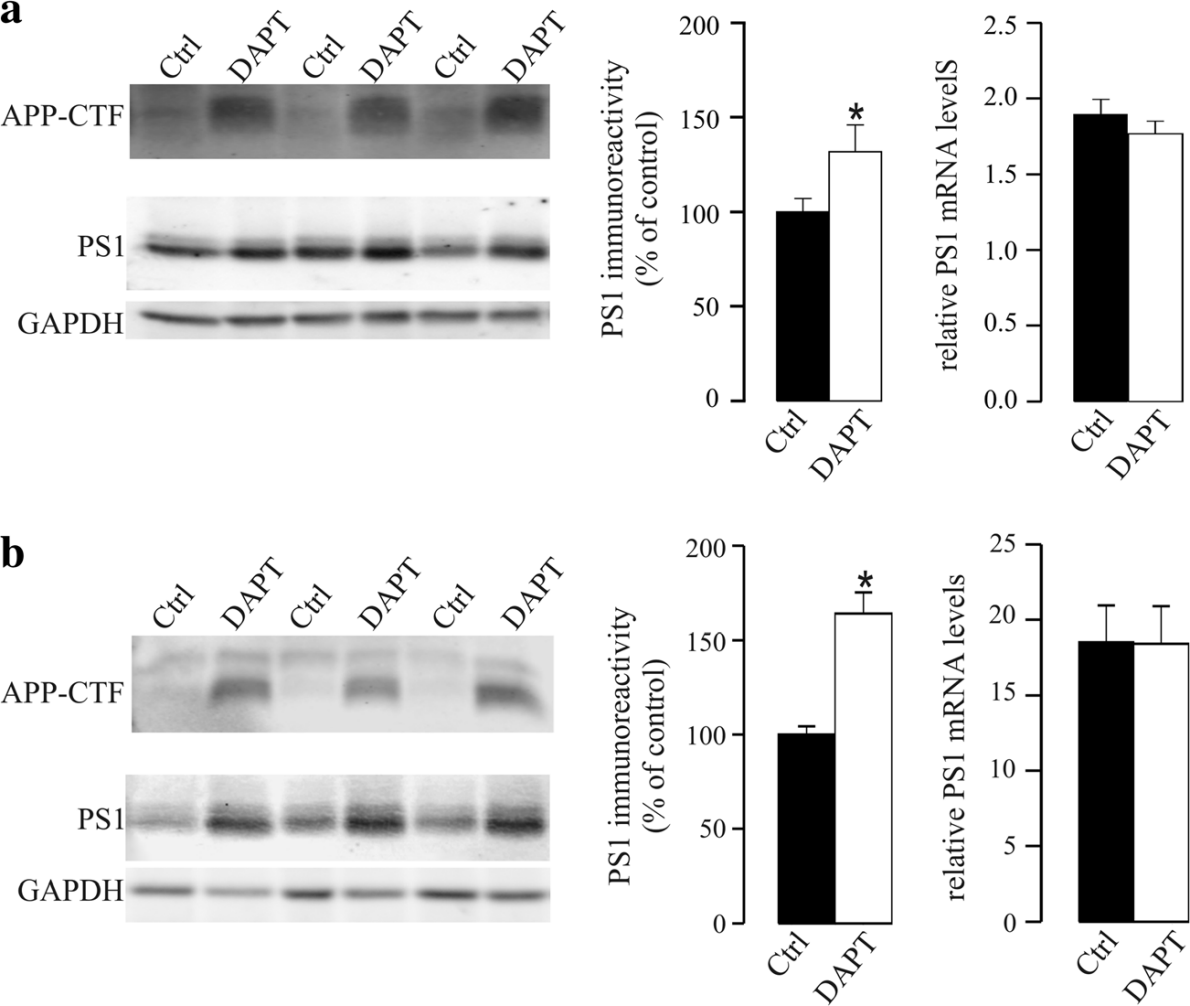
GSI DAPT treatment augments PS1 in SH-SY5Y cells and in mouse primary neurons. **a** SH-SY5Y cells were treated for 18 h (acutely) with DAPT (5 μM) or the vehicle alone (control; Ctrl). Cell extracts were probed with antibody C1-6.1, against the APP C-terminal, to demonstrate the accumulation of the APP-CTF in treated cells as a result of the inhibition of γ-secretase processing. Cell extracts were also probed for PS1 with an anti-N-terminal antibody. Equivalent amounts of protein were loaded in each lane, and GAPDH was used as a loading control. Representative blots and densitometric quantification of the immunoreactivity are shown. Relative expression of PS1 mRNA was also analyzed by qRT-PCR. Transcript levels were calculated by the comparative 2^−ΔCt^ method with respect to GAPDH cDNA. **b** Primary neurons were treated with DAPT (2 μM) or the vehicle alone (Ctrl) for four consecutive days. Cell extracts were probed for APP-CTF and PS1 and for GAPDH as a loading control. The densitometric quantification for PS1-NTF is shown, as well the relative mRNA levels of the PS1 transcript. The data represent the means ± SEM of at least *n* = 10 independent determinations (obtained from two independent sets of experiments): *p < 0.05

We addressed whether DAPT, a well-known GSI that targets PS1 and reduces Aβ in vivo [30], alters PS1 expression and protein levels in SH-SY5Y neuroblastoma cells. Exposure to DAPT (5 μM) for 18 h did not affect cell viability (*p* = 0.6), as evaluated by the MTS assay and in agreement with a previous study [31]. We first corroborated the efficiency of an acute 18-h treatment with DAPT (5 μM) to inhibit γ-secretase activity by measuring the accumulation of the APP-CTF in cell extracts (Fig. 1a). PS1 undergoes endoproteolytic cleavage as part of its maturation, generating N-terminal fragment (NTF) and CTF [32], with very little full-length PS1 detectable in wild-type cultured cells [33]. As expected, a predominant band of ~ 29 kDa that corresponded to the PS1-NTF was evident when immunoblots were probed with an anti-PS1-NTF antibody, with little or no full-length PS1. The amount of PS1-NTF was significantly higher in extracts from DAPT-treated cells (32 ± 14%, *p* = 0.03) relative to the untreated controls (Fig. 1a). Similarly, there was a significant increase in the other γ-secretase components (nicastrin, PEN2, and APH1) in DAPT-treated SH-SY5Y cells (Supplemental Fig. 1A). However, there was no parallel increase in the mRNA encoding PS1 (Fig. 1a) or the other γ-secretase subunits (Supplemental Fig. 1B), which remained similar in DAPT-treated and untreated SH-SY5Y cells.

Likewise, repeated DAPT treatment of mouse primary neuronal cultures grown for 2 weeks and then treated daily with DAPT (2 μM) over 4 days also augmented the amount of PS1 protein (64 ± 11%, *p* < 0.001; Fig. 1b), with unaltered mRNA levels (Fig. 1b). Again, no cytotoxicity was observed during the treatment (*p* = 0.4, as compared with cell viability in cells treated with vehicle). Hence, the change in PS1 content persisted when γ-secretase inhibition was maintained.

### Effects of APP-CTF Over-expression on PS1 in SH-SY5Y Neuroblastoma Cells

**Fig. 2:**
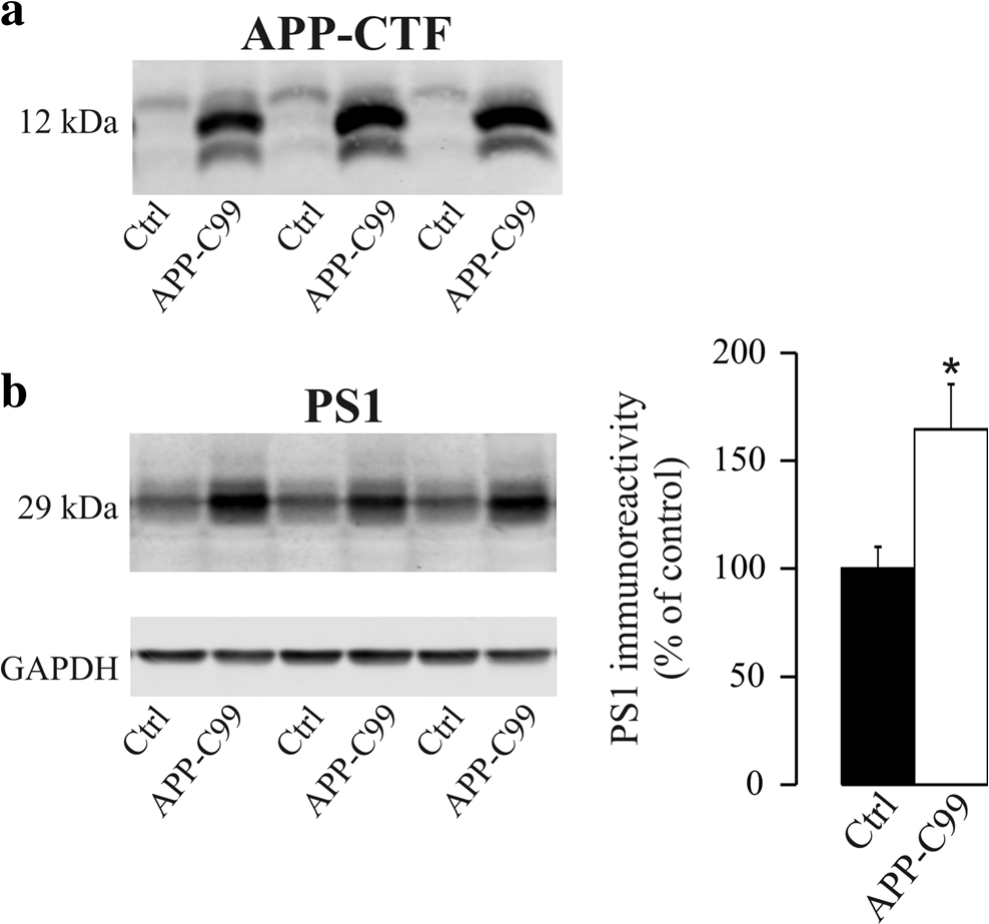
Effects of the modulation of APP-CTF expression on PS1 levels. SH-SY5Y cells were transfected with APP-C99 cDNA, the β-secretase-derived CTF of APP, or with a control vector (Ctrl). **a** Immunodetection of APP-CTF with the anti-APP C-terminal antibody C1-6.1 served to assess the efficiency of over-expression. The identity of the increased immunoreactive band was also tested with the 6E10 antibody, which recognizes an epitope present in the N-terminal of APP-C99 (not shown). **b** The immunodetection and densitometric quantification of PS1 immunoreactivity in transfected cells are shown. The data are presented relative to control cells, expressed as the means ± SEM of at least 12 independent determinations (obtained from two independent sets of experiments): *p = 0.007

Since APP-CTF accumulation is a consequence of γ-secretase inhibition, we tested whether increasing APP-CTF mediated the change in PS1 levels by transfecting SH-SY5Y cells with APP-C99 cDNA, the β-secretase-derived CTF of APP. More APP-CTF was evident in these cells following transfection (48 h; Fig. 2a), with APP-C99 over-expression producing a significant increase in the cellular PS1 content (65 ± 21%, *p* = 0.007; Fig. 2b).

### The GSI Avagacestat Alters the PS1 in Cultured Cells and Its Content In Vivo

**Fig. 3:**
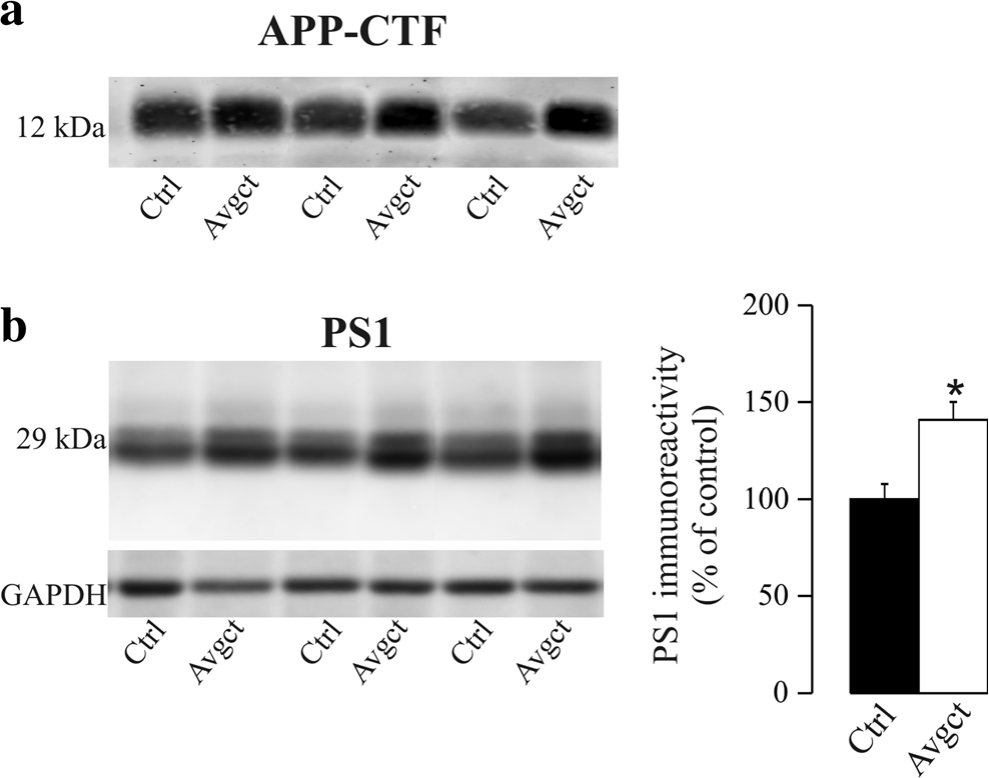
Increased PS1 levels in neurons treated with the GSI, avagacestat. Primary neurons were treated with avagacestat (2 μM, Avgct) or the vehicle alone (Ctrl), and the cell extracts were probed for **a** APP-CTF and **b** PS1. Representative blots and their densitometric quantification are shown. The data presented are relative to the Ctrl cells, expressed as the means ± SEM of at least ten independent determinations (obtained from two independent sets of experiments): *p = 0.007

Avagacestat is one of the first GSI that undergone clinical trials but discontinued development for AD because of a lack of efficacy at phase 2 trial [34–36]. Avagacestat selectively blocks the processing of APP substrates without notably affecting Notch processing [37, 38]. We analyzed the effect of avagacestat on PS1 in the primary neuronal cultures, where exposure to this GSI (2 μM) on four consecutive days increased the amount of PS1 relative to the controls exposed to the vehicle alone (41 ± 9%, *p* = 0.007; Fig. 3). There was no cell death in cultures treated with avagacestat, as evaluated by the MTS assay (*p* = 0.5).

**Fig. 4:**
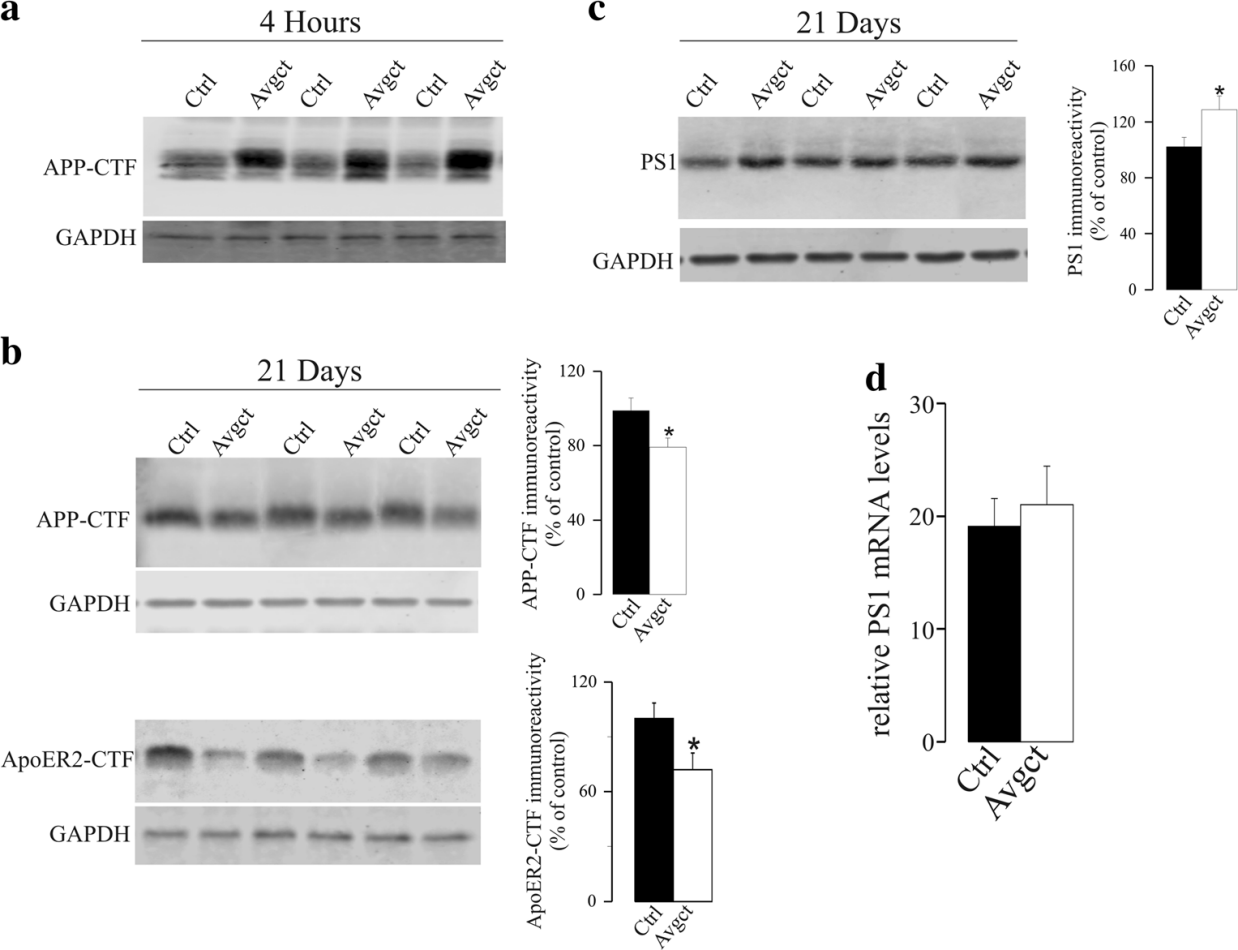
Effect of prolonged inhibition of γ-secretase by avagacestat on γ-secretase substrates and PS1 in the cortex of rats treated for 21 days. Rats were treated daily with the GSI avagacestat (40 mg/kg, Avgct) or the vehicle alone (control; Ctrl) for 21 days, and they were sacrificed 4 h after the last dose. **a** As a control of the effective γ-secretase inhibition by the GSI in the brain, APP-CTF levels (probed with antibody C1-6.1) were firstly evaluated in rats sacrificed 4 h after a single dose of avagacestat (*n* = 6 per group). **b** The levels of APP-CTF and ApoER2-CTF were estimated in rats treated with avagacestat for 21 days; representative blots and densitometric quantifications are shown. **c** PS1 levels were also evaluated in Western blots of the same brain hemi-cortex extracts. GAPDH was used as a loading control. **d** Relative PS1 mRNA was analyzed by qRT-PCR in the other rat hemi-cortices obtained after 21 days of treatment (*n* = 10 per group). The data are presented relative to the control rats and expressed as the means ± SEM (*n* = 10 per group): * *p* < 0.05 significantly different from the controls

Avagacestat was also administered orally to rats in a 40 mg/kg dose. In previous experiments in rats to which doses of 2–100 mg/kg avagacestat were used, a 40 mg/kg dose demonstrated significantly reduced Aβ in the brain, with no abnormalities detected [37, 39]. Acute treatment served to probe that avagacestat inhibits the processing of APP-CTF in treated rats, promoting their accumulation in animals treated with a single dose (Fig. 4a). We also tested whether avagacestat treatment increases PS1 in the brain of rats as part of a rebound effect, and we extended our analysis to include behavioral tests. When avagacestat (40 mg/kg) was administered orally to rats once daily for 4 days, there was apparently no effect on the amount of APP-CTF in the brain after treatment and PS1 levels remained unaltered (Supplemental Fig. 2). Conversely, treatment for 21 days significantly diminished the APP-CTF in the brain (79 ± 5%, *p* = 0.005; Fig. 4b). This unexpected decrease in APP-CTF, after prolonged GSI treatment, prompted the analysis of the levels of other γ-secretase substrates. ApoER2, a liporeceptor for ApoE/Reelin, is also a γ-secretase substrate [31], and a significant decrease in ApoER2-CTF (72 ± 9%, *p* = 0.03; Fig. 4b) was also detected in rats exposed to avagacestat, relative to the control rats. The increase in the rate of processing of γ-secretase substrates, APP-CTF and ApoER2-CTF, paralleled with an increase in PS1-NTF (29 ± 9%, *p* = 0.008: Fig. 4c). Again, the avagacestat-induced increase in PS1 protein was not paralleled by an increase in its mRNA transcripts (Fig. 4c).

**Fig. 5:**
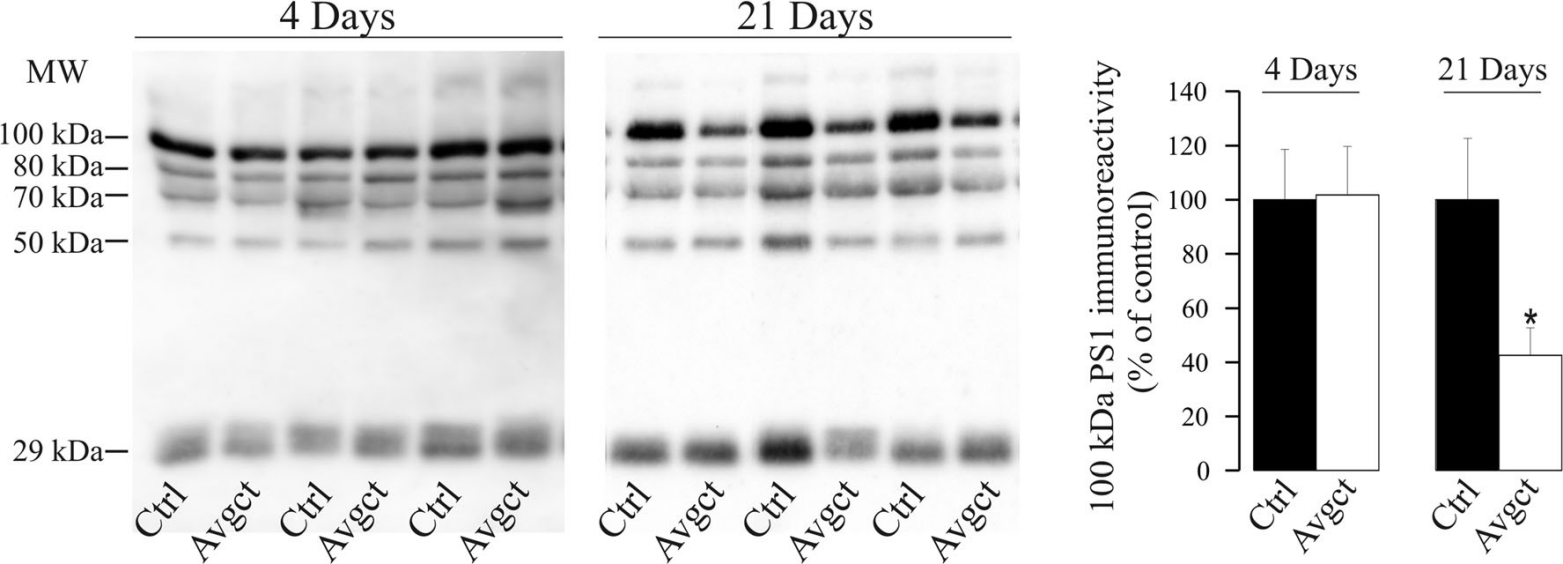
Effect of avagacestat on PS1 levels in CSF of rats treated for 4 and 21 days. Rats were administered avagacestat (40 mg/kg, Avgct) or the vehicle alone (Ctrl) daily over 4 or 21 days. Soluble PS1 complexes were also evaluated in Western blots of CSF samples from Avgct-treated and control rats (*n* = 7 per group). CSF-PS1 complexes were detected with and N-terminal antibody, which predominantly recognized stable complexes of approximately 100 kDa, together with less abundant 80-, 70-, and 50-kDa complexes, as well monomers of 29 kDa. Previous studies indicated that these CSF-PS1 complexes represent aggregates of PS1-NTF and CTF [40, 41]. The densitometric quantification of the major CSF-PS1 100-kDa complex is shown. The data are presented relative to the control rats, expressed as the means ± SEM: *p < 0.05

We recently demonstrated the presence of heteromeric PS1 complexes in human and rodent CSF (CSF-PS1), the proportion of such stable, large molecular mass complexes being associated to AD status [40, 41]. In Western blots probed with an antibody against the PS1-NTF, predominant bands of approximately 100, 80, and 70 kDa were detected, corresponding to CSF-PS1 SDS-stable complexes previously characterized [40], as well a 29-kDa band corresponding to monomeric PS1-NTF. Unexpectedly, the immunoreactivity for the 100-kDa complexes diminished in 21-day avagacestat-treated rats relative to the control rats (57 ± 10%, *p* = 0.03; Fig. 5), whereas no notable changes were observed in rats treated for 4 days with avagacestat (Fig. 5).

**Fig. 6:**
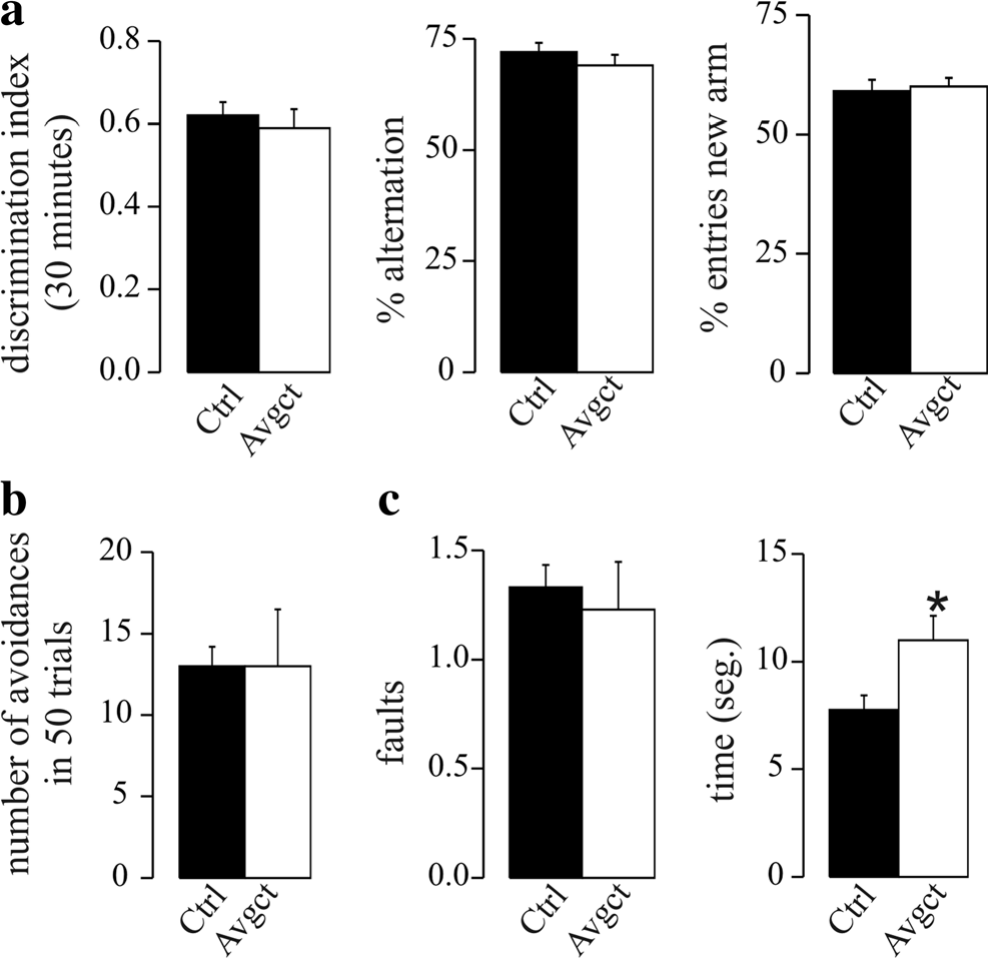
Results of the behavioral tests in rats treated 21 days with avagacestat. **a** Novel spatial recognition memory in the Y-maze in rats treated with avagacestat for 21 days (Avgct) and in the vehicle-treated controls (Ctrl). The time spent in each arm was recorded in order to calculate the discrimination index after a 30-min inter-trial interval. **b** Result of the active avoidance test documenting the number of attempts made to avoid the foot shock. **c** Beam walking test in which the number of slips and the latency to cross were scored. The values are the means ± SEM (*n* = 10 for each group): *p < 0.05

Finally, we assessed potential behavioral, memory, and learning changes in rats treated for 21 days with avagacestat using the novel spatial recognition memory, the active avoidance, and the beam walking tests. Avagacestat-treated animals displayed no differences in the novel spatial recognition memory in the Y-maze, with similar discrimination between arms, nor delayed alternation, when compared to the control rats (Fig. 6a). We also observed similar abilities of avagacestat and vehicle-treated rats to learn the active avoidance task and avoid the aversive event (Fig. 6b). However, while avagacestat-treated rats did not display any alterations in the ability to cross a round beam, revealing no gross motor deficits, significant differences were detected in the latency time to cross the beam, probably indicating higher levels of anxiety (Fig. 6c).

## Discussion

The possibility that levels and activities of secretases are affected in the brain of AD subjects has been studied intensively [5, 8]. However, whether their inhibition by GSIs can induce persistent compensatory changes in the brain has yet to be addressed. It is known that neurotransmitter transporters potentially undergo alterations to gene transcription, mRNA translation/stability, post-translational, protein trafficking, cytoskeletal interactions, and oligomerization in response to chronic drug administration [42]. Indeed, an upregulation of proteins targeted by pharmacological inhibition has also been documented [18–20]. Here, we demonstrate that GSIs can induce a feedback mechanism that results in accumulation of PS1 in different cell models. A similar elevation of brain PS1 was identified in 21-day avagacestat-treated rats, which also displayed an increasing rate of processing of the γ-secretase substrates APP-CTF and ApoER2-CTF, indicative of a rebound effect. These effects could be related to the reported failure of GSIs to achieve long-term Aβ regulation and their contribution to rather than the palliation of the AD pathology.

We found an increase in PS1 after a single day of DAPT administration to SH-SY5Y cells. Similar results were obtained in primary neuronal cultures treated for 4 days with DAPT and in rats treated for 21 days with avagacestat. The increase in PS1 protein was not paralleled by changes in PS1 mRNA content, indicating that this increase is not mediated by transcriptional upregulation. At present, the mechanism by which PS1 levels are enhanced by GSI administration remains unknown. Interestingly, over-expression of the β-secretase-derived APP fragment C99 could also mediate an increase in PS1. There is evidence that the accumulation of APP-C99 may be directly implicated in neurodegeneration and cognitive alterations [43]. Previous evidences indicate that excess in other γ-secretase substrates can compromise γ-secretase catalytic activity, being accompanied by an increase in PS1 levels [21]. Remarkably, it has been demonstrated that accumulation of APP-C99 can cause an impaired lysosomal-autophagic function [44]. Hence, it seems desirable to investigate whether an excess of γ-secretase substrates may result in transient stabilization of PS1/γ-secretase substrate complexes, interfering in the effective clearance of PS1. Similarly, the stabilization of PS1/GSI complexes during sustained γ-secretase inhibition could interfere in the clearance/turnover of PS1. Indeed, decreased intracellular clearance of PS1 may also reflect the reduction of the CSF-PS1 complex levels, although how PS1 reaches the CSF is unknown.

In this context, it has been demonstrated that changes in PS1 ubiquitination can alter cellular levels of PS1 and other γ-secretase subunits, leading to an alteration in the metabolism of APP [45, 46]. Therefore, a chronic treatment with GSIs may cause a sustained accumulation of PS1 leading a rebound effect with gain in γ-secretase activity. In this regard, although avagacestat has demonstrated effect in the accumulation of APP-CTF (acute treatment in rats), prolonged exposition to the GSI (21 days of treatment in rats) has lead to PS1 accumulation. An increase in PS1 levels, even maintained in GSI treatment, could result in an increasing rate of substrate processing during the oscillations in the effective inhibitory concentration of the drug, derived of the half-life and QD dosing.

Moreover, other alternatives are suitable. There are subtle differences in the subcellular accumulation of APP-CTF in PS1-deficient cells, with no obvious redistribution of the full-length protein [47]. Distinct subcellular locations of PSs have been shown to contribute to substrate specificity [48], and changes in the subcellular distribution of BACE1 induced by Aβ oligomers have been related to the pathogenesis of AD [49]. In brief, both post-translational and turnover/degradation mechanisms may participate in the pernicious response to GSI and deserve investigation. Moreover, we cannot discard that other enzymes distinct from PS1, or acting in parallel, could be involved in the rebound effect, with an increased rating of γ-secretase substrate processing during prolonged inhibition.

Chronic inhibition of PS1 with GSI has led to toxic side effects in clinical trials [37, 50, 51]. These adverse effects were thought to be related with the regulation of Notch activity by γ-secretase, a protein that is important for cell-to-cell communication and that has also been implicated in cancer [52]. Toxic side effects have been noted in clinical trials conducted with *Notch-sparing* GSIs as well as *non-selective* GSIs, although the true selectivity of the former is not clear [8]. Indeed, dozens of additional substrates for γ-secretase have been identified and, thus, non-selective GSIs would probably interfere with multiple cellular events [53, 54]. Currently, clinical trials with semagacestat (LY450139), an earlier-generation GSI that does not discriminate well between APP and Notch, have been discontinued, similar to clinical trials with avagacestat. Furthermore, the development of another Notch-sparing GSI, begacestat (GSI-953) [55], has also been discontinued for reasons that are not clear (discussed in De Strooper and Chávez-Gutiérrez [56]).

The therapeutic effect of GSIs appears to be transient, and the possibility of decelerating or halting cognitive deterioration also falls below expectations. At 2 years, no significant differences were observed in key clinical outcome measures in an avagacestat phase 2 trial, yet progression to dementia was more frequent in the prodromal AD cohort vs the observational cohort [36]. Similarly, semagacestat made AD patients cognitively worse in a phase 3 trial [57]. In Tg2576 mice, a 1-day treatment with two GSIs significantly ameliorated cognitive deficits (acute effects) but these effects disappeared when an 8-day treatment schedule was employed. Indeed, prolonged treatment with GSIs impairs spatial working memory and cognitive function [58]. In our study, an augmented latency time in the beam walking test in wild-type rats treated for 21 days with avagacestat suggests that some behavioral issues are affected by GSIs. This phenomenon is consistent with the dampening of initiative and the anxiety that are common neuropsychiatric features of AD [59, 60]. Interestingly, the conditional double presenilin knockout mice has observably altered anxiety-like behavior [61], and less anxiety is also displayed by transgenic mice expressing mutants PS1-A246E [62] and PS2-N141I [63]. An association of PS1 with altered anxiety-like behavior has been suggested [64] and is worthy of further investigation. The subtle alterations in behavioral tests in wild-type rats are inconclusive since we did not use an animal model with an impaired condition, and nor did we demonstrate a direct association between altered anxiety-like behavior and increased brain PS1 levels. However, we speculate that part of the impairment observed in clinical trials involving GSI use on humans and in chronically treated animals could be due to rebound increases in PS1.

Although simple in concept, the validation of amyloid drug targets, and specifically that of GSIs, has proved complex in practice. Earlier studies indicated that the acute oral administration of DAPT to APP_V717F_ transgenic mice reduces the Aβ in the brain [30]. The use of canine [65] and non-human primate [66] models also served to demonstrate that GSIs decrease the Aβ peptides in the CSF. However, it is well established that the levels of AD CSF diminish when there is an increase in brain deposition of Aβ. Thus, changes in CSF-Aβ are unlikely to provide significant information about therapies aimed at reducing Aβ production, and a lowering of CSF-Aβ levels is unlikely to be a suitable measure of target engagement [67].

In this regard, acute administration of avagacestat robustly reduces CSF Aβ40 and Aβ42 levels similarly in rats and dogs [39]. Moreover, the administration of a single dose of avagacestat to healthy humans, as well over a 28-day schedule, also markedly decreases Aβ40 and Aβ42 concentrations in the CSF [68, 69]. However, exploratory CSF amyloid isoforms displayed a dose-dependent but not significant reduction in a small subset of patients in a phase 2 trial, and while well tolerated, lower doses did not affect the Aβ40 and Aβ42 levels in treated patients [35]. Similarly, earlier studies with semagacestat in volunteers indicated unchanged levels of CSF-Aβ [70], although in another study, single oral doses of semagacestat appeared to decrease Aβ levels in the CSF of healthy volunteers [71]. No significant reduction in CSF Aβ42 or Aβ40 level was detected in a phase 2 safety trial [72], a finding verified by mass spectrometry analysis of the same samples [73]. Instead, an increase in shorter Aβ peptides (Aβ1–14, Aβ1–15, and Aβ1–16) was identified, probably due to increased substrate availability (APP-C99) for α-secretase [73]. Interestingly, semagacestat produced a decrease in plasma Aβ concentrations in a 6-h interval following drug administration, returning to baseline and then transiently increasing the Aβ concentrations [13]. It was suggested that semagacestat might lower Aβ at high concentrations but cause Aβ elevation at low concentrations [15]. A structurally related γ-secretase inhibitor, LY-411575, also elevated plasma Aβ40 and Aβ42 in Tg2576 mice [14]. A biphasic activation-inhibition dose-response curve for GSIs was proposed to explain these changes in Aβ secretion [74]. However, these changes may also be indicative of a transient overshooting or rebound effect, since an increase in plasma Aβ40 and Aβ42 has been described in Tg2576 mice chronically treated with DAPT [12].

Here, we addressed the efficiency of GSIs to inhibit PS1 by assessing changes in the cellular γ-secretase substrate APP-CTF. As expected, the accumulation of APP-CTF served to assess the inhibitory effect of DAPT on PS1 in cellular models and also that of avagacestat. Accordingly, we were able to detect accumulation in the brain levels of APP-CTF in acutely treated rats (sacrificed 4 h after a single dose). However, sustained inhibition of γ-secretase activity over 21 days revealed decreased APP-CTF levels, suggesting that the consolidation of higher PS1 levels in reaction to chronic inhibition results in an increase in γ-secretase activity, at least in the intervals between GSI administration. The consolidation of higher levels of PS1 might indiscriminately affect all γ-secretase substrates, such as ApoER2 and others, further exacerbating the AD pathology. Interestingly, administration of GSIs increased APP-CTF in H4 cells over-expressing APP, although this increase was unexpectedly attenuated at high concentrations [58]. Elsewhere, APP-C99 levels increase in CHO cells co-expressing APP and PS1 relative to cells expressing APP alone, and PS1 can stabilize APP-CTF independent of γ-secretase activity [75]. Hence, the relationship between the substrate and the catalytic enzyme appears to be more complex than might at first appear.

PS1 also participates in other cell functions [76, 77], and therefore, the increase in PS1 after GSI administration may influence distinct cellular effects, even if this subunit is not-catalytically active. In this regard, PS1 has been implicated in the physiological maturation and glycosylation of several key proteins implicated in AD, such as nicastrin [78], BACE1 [79], acetylcholinesterase [80], and others, including APP [81]. Hence, the over-expression of either the wild-type or mutant PS1 disturbs glycoprotein processing [82]. Further research will be needed to clarify the influence of increased PS1, under prolonged GSI administration, in the role of PS1 in their non-proteolytic functions, and possible interference with the therapeutic response.

## Conclusions

We show here that administration of GSIs result in a rebound increase in PS1 levels in cellular and animal models, which must be taken into consideration when using such compounds in AD therapy. Indeed, our results indicate that the effect of GSI inhibitors on APP processing failed to have a long-term effect in treated rats, possibly due to the persistent PS1 elevation in reaction to chronic inhibition.

The outcomes of the clinical trials with GSIs have been disappointing, although this may not represent the end of the development of these drugs to treat AD. The data presented here indicate that the therapeutic benefits of GSIs and related drugs should continue to be explored, or at least, we can extract information that will help understand the failure of GSIs in AD trials [83]. Hence, synthesizing new GSIs that distinguish strongly between APP and Notch may serve to lower the required dose, yet it still might not solve the unexplained and unexpected problem of the facilitation of toxic side effects and the AD-derived pathogenesis. The failure of GSIs in clinical trials highlights the need for a systematic re-examination of γ-secretase biology, including further characterization of the mechanisms related to the response to chronic inhibition. Elucidating the mechanisms involved, the complex self-regulation of γ-secretase is also important to optimize therapies based on γ-secretase modulation. A potent inhibition/modulation of secretase activities will result in the unbalanced generation of proteolytic fragments of APP (and fragments from other substrates), which could determine a self-regulatory response that will require further analysis for new secretase inhibitors/modulators designed to specifically inhibit the Alzheimer process.

In this regard, γ-secretase modulators (GSMs), which only block the γ-secretase cleavage of APP to generate the Aβ42, with no changes in the production of total Aβ, were also noticed to have negative outcomes [84]. The clinical development of BACE1 inhibitors is also being intensely pursued, and several promising BACE1 inhibitors have entered human clinical trials [85], but a sign of toxicity forced to stop the earliest trials (discussed in Lahiri et al. [86]). For a successful development of new secretase inhibitors/modulators, it is needed to better understand the cellular response to the sustained inhibition/modulation of the secretase activity.

Despite its enzymatic capacity, γ-secretase activity appears tightly regulated by many cellular components, including its own subunits, modulatory partners, and substrates, as well as by an array of cellular events [87]. Furthermore, GSIs are presently explored in clinical trials as potential therapeutic agents in cancer, targeting Notch, although a number of mechanism-based adverse events again emerge [88]. As the therapeutic benefits of GSIs and related drugs continue to be explored, a better understanding of the response of PS1 to chronic inhibition will become more necessary.

## Electronic supplementary material

ESM 1 (PDF 867 kb)
