# Supplementary material for: Inhibition of γ-Secretase Leads to an Increase in Presenilin-1
Source: Mol Neurobiol. 2017 Aug 16;55(6):5047–58. doi: 10.1007/s12035-017-0705-1 (PMC5948247; doi:10.1007/s12035-017-0705-1)
Supplement: Supplementary file 1 — (PDF 867 kb) [file 12035_2017_705_MOESM1_ESM.pdf]

Supplementary Figures

Inhibition of γ-secretase leads to an increase in presenilin-1

Aitana Sogorb-Esteve[1,2]; María-Salud García-Ayllón[1,2,3]*; Marta Llansola[4];

Vicente Felipo[4]; Kaj Blennow[5,6]; Javier Sáez-Valero[1,2,]*

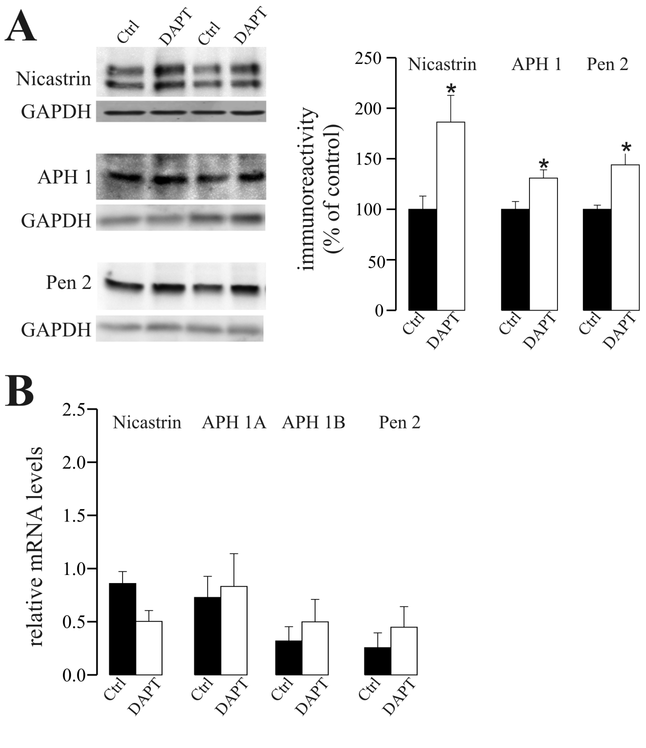

***Supplemental Figure 1*. GSI DAPT augments γ-secretase subunits in SH-SY5Y cells.** (**A**) SH-SY5Y cells were treated for 18 h (acutely) with DAPT (5 μM) or the vehicle alone (control; Ctrl), as indicated in Fig. 1. Cell extracts were probed for subunits of the γ-secretase complexes: nicastrin, PEN2, and APH1 (with an antibody that recognizes both the APH1A and APH1B forms). Equivalent amounts of protein were loaded in each lane and GAPDH was used as a loading control. Representative blots and densitometric quantification of the immunoreactivity are shown. For nicastrin the upper band is the mature form [Kimberly *et al* 2003] [$] and the band quantified. (**B**) Relative expression of nicastrin, PEN2 and APH1 (homologs APH1A and APH1B) mRNA was analyzed by *q*RT-PCR. Transcript levels were calculated by the comparative $2^{-\Delta Ct}$ method with respect to GAPDH cDNA. The data represent the means ± SEM of at least n=10 independent determinations (obtained from two independent set of experiments): *$p < 0.05$.

[$] Kimberly WT, LaVoie MJ, Ostaszewski BL, Ye W, Wolfe MS, Selkoe DJ (2003) Gamma-secretase is a membrane protein complex comprised of presenilin, nicastrin, Aph-1, and Pen-2. Proc Natl Acad Sci USA 100:6382-6387.

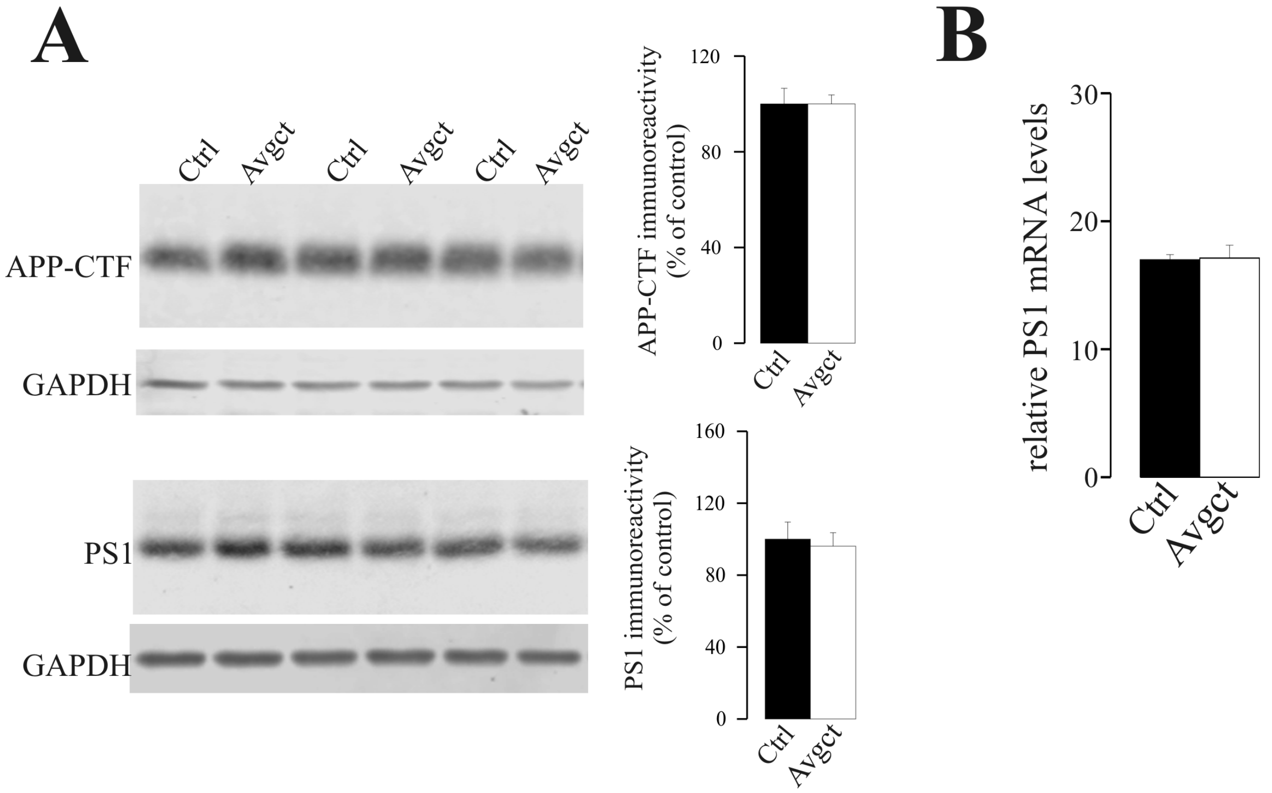

Supplemental Figure 2. **Effect of avagacestat on APP-CTF and PS1 levels in the cortex of rats treated for 4 days**. Rats were treated daily with the GSI avagacestat (40 mg/kg, Avgct) or the vehicle alone (control; Ctrl) for 4 days (n= 10 per group), and they were sacrificed 4-5 hours after the last dose. (**A**) The APP-CTF and PS1 were evaluated in Western blots of extracts from brain hemi-cortices. Representative blots probed for APP-CTF and PS1, and the densitometric quantifications, are shown. GAPDH was used as a loading control. (**B**) Relative PS1 mRNA was analyzed by *q*RT-PCR in rat hemi-cortices. The data are presented relative to the control rats, expressed as the means ± SEM: *$p < 0.05$.
